# Contribution of community-based organizations in the improvement of Joint United Nations Program on HIV and AIDS 90-90-90: case of the Yaoundé Central Hospital

**DOI:** 10.11604/pamj.2023.45.173.38466

**Published:** 2023-08-21

**Authors:** Charles Kouanfack, Marie Mvilongo, Sylvain Zemsi, Lauriane Fomete, Clarisse Mapa-Tassou, Alain Djam, François Zambou, Jerome Ateudjieu, Pierre Joseph Fouda

**Affiliations:** 1 Faculty of Medicine and Pharmaceutical Sciences, University of Dschang, Dschang, Cameroon,; 2 Yaounde Central Hospital, Yaoundé, Cameroon,; 3 Faculty of Medicine and Biomedical Sciences, University of Yaoundé, Yaoundé, Cameroon

**Keywords:** Contribution, community-based organization, improvement, 90-90-90, Joint United Nations Program on HIV and AIDS

## Abstract

Community-based organizations (CBOs) are one of the initiatives implemented in Cameroon to improve access to antiretroviral treatment and influence retention in treatment centers. Despite its importance in the decongestion of patients in health facilities, we do not have data to evaluate the overall impact of these organizations. We conducted a two-part observational study. The first part was a descriptive cross-sectional study, where we included patients screened and initiated on anti-retroviral treatment (ART) either by the approved Treatment center (ATC) of Yaoundé Central Hospital (YCH) or by any of our CBOs in 2020. Then, the second part was a retrospective cohort-type study including patients from the 2015 cohort followed up from 2018 to 2020 in order to assess viral load suppression. As regards the first “90”, 7,234 screening tests were performed by CBOs in 2020 out of the 28,302 screening tests registered at the YCH, giving a contribution of 25.6%. From the 7,234 screening tests performed by CBOs, 314 people had an HIV-positive result and 230 (73.34%) were linked to ART through CBOs. From the 28,302 screening tests performed at YCH, 1,089 people had an HIV-positive test, and only 354 (32.50%) were linked to ART, giving a significant difference in the link to ART (P-value < 0.00). Concerning the 3^rd^ ''90'', the viral load suppression rates were respectively in CBOs and at YCH of (95.12% vs 90.54%, RR= 0.51; P-value= 0.27 at 12 months); (95.96% vs 95.34%, relative risk (RR)= 0.85; P-value= 0.81 at 24 months); and (96.91% vs 94.15%, RR= 0.52; P-value = 0.24 at 36 months). In conclusion, we say that the follow-up of patients living with HIV in the community does not negatively affect the evolution of the disease as one might think.

## Introduction

In 2014 the Joint United Nations Program on HIV and AIDS (UNAIDS) set up a response plan to HIV infection under the name 90-90-90 with the objective that, by 2020, 90% of all people with HIV will be diagnosed, 90% of all people diagnosed will be on treatment, and 90% of people on treatment will achieve viral suppression [1]. In order to achieve this ambitious target, each country needed to focus simultaneously on HIV testing/diagnosis, treatment, and viral suppression [2]. Five years after the implementation of this strategy, UNAIDS in its 2019 annual report reported that globally, 84% [68-98%] of people knew their status, 73% [57-88%] of these people had access to treatment and 66% [53-79%] had a suppressed viral load [3]. In Cameroon, the National Committee's annual report on sexually transmitted infections (STI) control activities showed statistics below these objectives, with nearly 83.8% of patient living with HIV (PLHIV) who knew their status, 73.6% of identified PLHIV who were on ART. Only 34.4% of people on ART had had a viral load test, of which 88.0% had a suppressed viral load. However, in the same year data reported by some countries with very different economic contexts, such as Australia or Botswana, showed that it was possible to achieve these objectives through concerted efforts, in particular by giving priority HIV testing to communities and setting up therapeutic relay initiatives [4]. To improve national performance, the Ministry of Public Health of Cameroon decided to establish in 2017, associations of people living with HIV or affected by HIV, based in the community (CBO), whose existence was recognized by the competent administrative authority [2]. These organizations, therefore, aim to reduce the number of patients in some approved treatment centers (ATC) and care units for PLHIV. In addition, they will help reduce stigma, improve geographical and economic hindrances that had been identified as factors for inadequate follow-up [5]. To date, there are approximately 100 CBOs throughout the territory following approximately 20,000 patients living with HIV (PLHIV), representing about 6.1% of the national HIV cohort [6]. However, we lack accurate data to evaluate the overall effectiveness and impact of these organizations on reaching and achieving the UNAIDS 90-90-90 targets. We, therefore, decided to conduct this study to assess the contribution of these community-based organizations in improving testing, treatment linkage, and viral load suppression.

## Program evaluation

**Study design:** we carried out a two-part observational study in the various community-based organizations and in the approved treatment center (ATC) of the Yaoundé Central Hospital between July 2020 and July 2021. In the first part, it was a descriptive study including patients screened and put on ART thanks to the six CBOs of the Central Hospital of Yaoundé in 2020. In the second part, it was an analytical study retrospective cohort type.

**Setting:** a community-based organization is an association of people within a community, united for the defense of common interests with common goals well-defined. In Cameroon, Community-based organizations of people living with HIV were recognized by the authorities in 2017, with the objectives of promoting HIV awareness, giving psychosocial support to newly diagnosed patients, providing antiretroviral (ARV) treatments to stable patients defined by the ATC, and preventing new infections through frequent screening in the community [7]. They also play a great role in identifying patients the follow not taking their ARV properly, and in managing their drug supplies received when living from approved therapeutic centers (ATC). These organizations are directly under the supervision of approved therapeutic centers and help monitor people living with HIV. The approved therapeutic center has the responsibility to identify and select patients who meet the eligibility criteria and suggest that they continue follow-up and obtain their ARV from these CBOs who are generally within a radius of the patient´s house. For a patient to be transferred to a CBOs for follow-up, he has to be at least 20 years old, have been under treatment for more than 12 months, should be clinically stable with an undetectable viral load and/or a CD4 count > 500 cell/mm^3^, should be free of visible signs of an opportunistic infection, should not be pregnant, should consent to the CBO initiative and should be on first-line ARV treatment [7]. When a patient fills these criteria, he is eligible to live in the approved treatment center (ATC) for any CBOs close to his living area [7]. The CBOs have the responsibility to collect the necessary drugs from the national program pharmacy according to the number of patients they are following and assure dispensation to them [7]. Community Based Organization perform screening test, and whenever a new patient has a positive HIV-test, the CBO ensure that the patient is transferred to the relevant ATC for treatment initiation.

**Participants:** given the dual aspect of our study, the selection criteria were established as follows: i) for the descriptive part, we included all patients followed by community-based organizations affiliated with the Central Hospital of Yaoundé (approved therapeutic center) and excluded all community based organizations not providing screening tests activities; ii) for the analytical component, we included in the exposed group patients from the 2015 cohort who were stable and referred in 2017 to the CBOs for ART supply, and in the non-exposed group, patients from the 2015 cohort who had CBOs criteria but were maintained on ART in 2017 at the Yaoundé Central Hospital. We excluded all patients whose files were incomplete.

**Data sources and measurements:** a grid was developed to collect sociodemographic characteristics, screening test results, date of ART initiation, treatment protocol, viral load results, testing, treatment linkage, and viral load suppression. The patients were matched in the two groups according to gender, age, and treatment protocols in order to limit bias in the analyses.

**Study size:** we realized a consecutive and exhaustive sampling in those six CBOs and at the ATC of the Yaoundé Central Hospital for our study period and included all patients meeting our defined inclusion criteria.

**Statistical methods:** descriptive statistics (i.e. screening test results, viral load results, treatment linkage, and viral load suppression) collected at baseline were used to describe participant characteristics. Data analysis was done using EPI Info 7.2.2.6 software, the significance threshold was considered statistically significant at (P-value <0.05). We evaluated associations between categorical variables using the Chi^2^ test or the exact Fisher test as relevant. Associations between continuous variables and categorical variables were evaluated using student's test after checking the assumptions of normality and equality of variance.

**Ethical and administrative considerations:** the research protocol received administrative authorization from the Management of the Central Hospital of Yaoundé and ethical clearance from the Institutional Ethics Committee Board of this same institution. The standard measures necessary to guarantee the confidentiality of the information collected in the files have been taken. Only the identification numbers were entered on the collection sheets and access to the data was secured by an encrypted password.

## Results

**Contribution of community-based organization in the first “90” of UNAIDS:** in 2020, 7 234 people were screened by the CBOs out of a total of 28 302 people screened in the entire ATC of the Central Hospital of Yaoundé, giving a contribution of 25.6% in the improvement of the first 90. About the sociodemographic characteristics of the people screened, the average age was 32 ± 13 years, and the male sex was the most represented with 61.5% with a sex ratio of 1.6 (Table 1).

**Table 1:** distribution according to age and sex of people screened for HIV in 2020

Variables	Number (n=28302)	Percentage (%)	CI à 95%
**Age**			
0-14	1301	4.6%	[4.4- 4.9]
15-29	**12638**	44.7%	[44.1-45.2]
30-44	9979	35.3%	[34.7-35.8]
45-59	1352	4.8%	[4.5-5.0]
60+	3032	10.71%	[10.4-11.1]
**Sexe**			
Male	**17410**	61.5%	[37.9-39.0]
Female	10892	38.5%	[61.0-62.1]

CI: confidence interval

**Contribution of community-based organization in the second “90” of UNAIDS:** of the 28 302 people screened in 2020, we had 1 403 new cases of confirmed HIV infection, that is 314 cases screened by the CBOs and 1 089 by the ATC. Regarding the link to treatment, the CBOs succeeded in putting 230 people screened out of 314, giving 73.2%, initiation on treatment, while the ATC was able to put 354 people screened out of 1089, giving 32.5% initiation on treatment with a P-value < 0.00. (Table 2). Regarding the sociodemographic characteristics of the people under treatment, the average age of the patients was 35 ± 10 years. The female sex was the most represented with 68.2% of people initiated, with a male/female sex ratio of 0.46.

**Table 2:** contribution of CBOs in the second ''90'' of Joint United Nations Program on HIV and AIDS

Variables	Number	Percentage (%)	P-value
Patients initiated on treatment by CTA	354	32.5	
Patients initiated on treatment by CBO’s	230	73.24	0.00

ATC: approved treatment center: CBO: community based organization

**Contribution of community-based organization in the third “90” of UNAIDS:** we did not find a significant difference in the rapid suppression of viral load in patients followed in the community and at the ATC of Yaoundé Central Hospital. However, the viral load achieved after 12, 24 and 36 months of ART were respectively for CBO and ATC of Yaoundé Central Hospital of 37.1% vs 34.4% (RR= 0.51 [0.15-1.69]) after 12 months; 55.6% vs 26.7% (RR = 0.86 [0.27-2.76]) after 24 months and 70.2% vs 95.5% (RR = 0.52 [0.18-1.54]) after 36 months (Table 3).

**Table 3:** contribution of CBOs in the third ''90'' of Joint United Nations Program on HIV and AIDS

Variables	Modality	CBO	YCH	RR	CI à 95%	P-value
Viral load at 12 m	Yes	78	67	0,51	[0,15-1,69]	0,27
	No	4	7			
Viral load at 24 m	Yes	119	123	0,86	[0,27-2,76]	0,81
	No	5	6			
Viral load at 34 m	Yes	157	145	0,52	[0,18-1,54]	0,24
	No	5	9			

M: month; CBO: community-based organization; YCH: Yaoundé Central Hospital; RR: relative risk; CI: Confidence interval

## Discussion

The study we conducted aimed to determine the contribution of CBOs in improving the three UNAIDS “90s”. To do this, we conducted a two-part study for 12 months in the ATC of the Central Hospital of Yaoundé and the CBOs attached to this ATC. In the first part, we describe the patients screened in each of these entities during the year 2020. In the second part, we analyzed data from a cohort of 2017 in order to evaluate and compare the effectiveness of CBOs to the YCH ATC. This study has the merit of being one of the first in our context to assess the role of CBOs in HIV care in Cameroon. However, we note limitations in this study, in particular, the poor matching of patients in the CBO and ATC groups due to the plurality of therapeutic protocols for the same patient. We also highlight the difficulty of finding all the necessary information in the different registers and archives. Regarding HIV screening at the Yaoundé Central Hospital, the CBOs carried out a quarter of tests carried out during the year 2020, giving 25.6%, which is inferior to data from other studies in a rural district of Malawi who found a community counselor contribution of 41% or 21 358 out of 52 510 HIV tests performed at the district and counseling sites [8]. Moreover, another study conducted in Zambia on the role of lay counselors in HIV counseling and testing services, showed that this contribution was low as they provide up to 70% of counseling and testing services in health facilities [9]. This could be explained by the fact that the screening activities in the CBOs are not yet well known by the populations in our context. Moreover, this activity is periodic and is only done at the request of a sponsor, and is not permanently financed. Regarding the initiation of antiretroviral treatment, we found poor results (less than half of newly diagnosed HIV patients were put on ART) at the Central Hospital of Yaoundé during the year 2020. Community-based organizations initiated more than a quarter of their HIV-positive patients into treatment linkage while YCH medical staff only linked a third of their HIV. These treatment enrollment rates are low in both groups according to UNAIDS, which recommends that 90% of PLHIV are linked to ART. Notwithstanding this finding, it is much better thanks to CBOs. These results are consistent with those of a systematic review and a study conducted in South Africa which demonstrated that ART initiation is facilitated through community interventions [10,11]. These results can be explained by the fact that the staff of community-based organizations being mostly counselors living with HIV, provide a set of services including the sharing of personal experiences of living with HIV; permanent support of patients in the realization and collection of results; assistance in alleviating real or perceived barriers to care. As regards to the suppression of the viral load after initiation of treatment, we studied the viral loads achieved at 12, 24, and 36 months of ART. Antiretroviral therapy at 12 months did not significantly increase (p-value=0.94, RR=0.51) viral load suppression in the community group compared to the ATC group. This result is equivalent to that from a study conducted in South Africa and Uganda which shows that viral load suppression at 12 months of ART is better in the community cohort, that is 74% and 63% in the hospital group [12]. The results at 24 months of ART were similar in the community and ATC cohorts, respectively 96.0% and 95.3% (P-value= 0.81, RR=0.86). These results are in line with studies conducted in Uganda which have proven that there is no significant difference between the results of suppressed viral load at 24 months (p-value= 0.12) [13]. These results can be explained by the problem of stigmatization that patients deplore in the hospital context, transport costs, and long waiting hours; unlike CBOs which are close to patients and operate at convenient times after hours, on weekends, and are flexible enough to accommodate the travel or mobility needs of quarterly ART refills.

## Conclusion

At the end of our study, whose objective was to determine the contribution of CBOs in the improvement of the three 90 of UNAIDS at Yaoundé Central Hospital, it appears that a quarter of patients screened at YCH were carried out thanks to CBOs in 2020. Community-based organizations have a better link to care with more than 2/3 of PLHIV on ART compared to ATC which only initiated less than a third. This study also shows that ART continuation in CBOs does not give poor viral load suppression results.
